# Monte Carlo simulation of digital photon counting PET

**DOI:** 10.1186/s40658-020-00288-w

**Published:** 2020-04-25

**Authors:** Julien Salvadori, Joey Labour, Freddy Odille, Pierre-Yves Marie, Jean-Noël Badel, Laëtitia Imbert, David Sarrut

**Affiliations:** 1 IADI, INSERM UMR 1254, Université de Lorraine, Nancy, France; 2 Département de médecine nucléaire et plateforme Nancyclotep, CHRU-Nancy, Université de Lorraine, Nancy, France; 3 CREATIS, Centre Léon Bérard, CNRS UMR 5220, INSERM U 1044, Université de Lyon; INSA-Lyon; Université Lyon 1, Lyon, France; 4 DCAC, INSERM UMR 1116, Université de Lorraine, Nancy, France

**Keywords:** Monte Carlo simulation, Digital photon counting, PET, NEMA

## Abstract

A GATE Monte Carlo model of the Philips Vereos digital photon counting PET imaging system using silicon photo-multiplier detectors was proposed. It was evaluated against experimental data in accordance with NEMA guidelines. Comparisons were performed using listmode data in order to remain independent of image reconstruction algorithms. An original line of response-based method is proposed to estimate intrinsic spatial resolution without reconstruction. Four sets of experiments were performed: (1) count rates and scatter fraction, (2) energy and timing resolutions, (3) sensitivity, and (4) intrinsic spatial resolution. Experimental and simulated data were found to be in good agreement, with overall differences lower than 10% for activity concentrations used in most standard clinical applications. Illustrative image reconstructions were provided. In conclusion, the proposed Monte Carlo model was validated and can be used for numerous studies such as optimizing acquisition parameters or reconstruction algorithms.

## Introduction

Positron emission tomography (PET) imaging has an essential role in modern medicine for both diagnostic and follow-up of oncology treatments [1]. PET technology has experienced tremendous improvements in performance over recent decades and new trends make use of silicon photo-multiplier (SiPM) detectors such as the Philips Vereos digital photon counting (DPC) PET/CT introduced in 2013, the GE Dicovery^TM^ MI PET/CT launched in 2016 and the Siemens Biograph Vision^TM^ PET/CT in 2018.

Precision in the location of the annihilation has been improved with the use of time-of-flight (TOF) information, which spatially constrains the location of the event on the line of response (LOR), increasing the signal-to-noise ratio (SNR) in the reconstructed image [2]. The TOF resolution improved with the use of SiPMs, due to their lower intrinsic timing resolution than conventional photo-multiplier tubes or avalanche photo-diodes, with a more compact electronic configuration [3]. Detecting and processing signals using digital SiPMs bypasses the need to treat analogous signals by a direct binary count of optical photons, reducing noise in the processed output. The Vereos DPC system has a 1:1 coupling between the crystal array and the SiPM array, which decreases uncertainty in the interaction position and ultimately improves the volumetric resolution on reconstructed images. The Vereos DPC system has been previously evaluated [4, 5] according to the National Electrical Manufacturers Association (NEMA) NU guidelines [6]. The spatial resolution, defined as the full width at half maximum (FWHM), was found to be 4.2 mm at the center of the field of view (FOV), the average sensitivity was estimated to be 5200 counts per second (cps)/MBq, and the peak noise equivalent count rate (NECR) was 153.4 kcps at an activity concentration of ^18^F of 54.9 kBq mL ^−1^ [4]. Several studies [7–9] have shown that the Vereos DPC can improve the image quality of PET images compared to analogous systems. Moreover, diagnostic confidence and accuracy for oncologic diseases is also improved [10–12].

Monte Carlo simulation is an important tool for PET imaging. It helps to design, optimize, and assess imaging systems; predict the performance; optimize acquisition parameters and reconstruction algorithms; and evaluate the effects of confounding factors in image quality. Several works have been proposed to simulate PET systems, notably via the GATE/Geant4 platform [13–15], such as [16–29] among others, but also with other softwares such as SimSET [30–33], PeneloPET [34] (Penelope), SORTEO [35], Eidolon [36] (MCNP), PETSIM [37], Geant4 [38], or GAMOS [39, 40]. Various PET imaging systems have been modeled and compared to experimental measurements, such as the ECAT HRRT [18] and EXACT HR+ [20, 21], Philips Allegro and GEMINI [19, 24], GE Advance and Discovery LS [23], and Siemens Inveon [29], Biograph 2 [21], Biograph 6 [25], and Biograph mcT [33]. However, to our knowledge, no Monte Carlo model of a SiPM-based PET system has been proposed and compared to experimental data.

In this work, the Philips Vereos DPC-PET system was modeled using the GATE platform and compared to measurements performed according to the NEMA protocols NU 2-2018 [6]. All comparisons were performed using listmode data in order to remain independent of the image reconstruction algorithm.

## Materials and methods

### Simulation physics parameters

The DPC-PET scanner was modeled with GATE 8.2 [13–15] using Geant4 10.5. Four simulations, corresponding to the four NEMA tests discussed, were carried out (see NEMA description in the “PET model validation” section). In all simulations, the physics list named emstandard_opt4 was used^1^. It contains the Geant4 most accurate standard and low-energy models for electromagnetic processes recommended for medical applications [41]. Range production cuts were set to 0.1 mm for electrons and photons in the whole geometry. In Geant4, it means that secondary particles are only created and tracked when their expected range in the current material is larger than this distance. No variance reduction technique was used. The radioactive sources of ^18^F were simulated by *β*^+^ sources with energy spectra parameterized according to the Landolt–Börnstein tables [13]. The number of primary particles was adapted for all simulations according to the NEMA protocol.

### PET scanner geometry

**Fig. 1:**
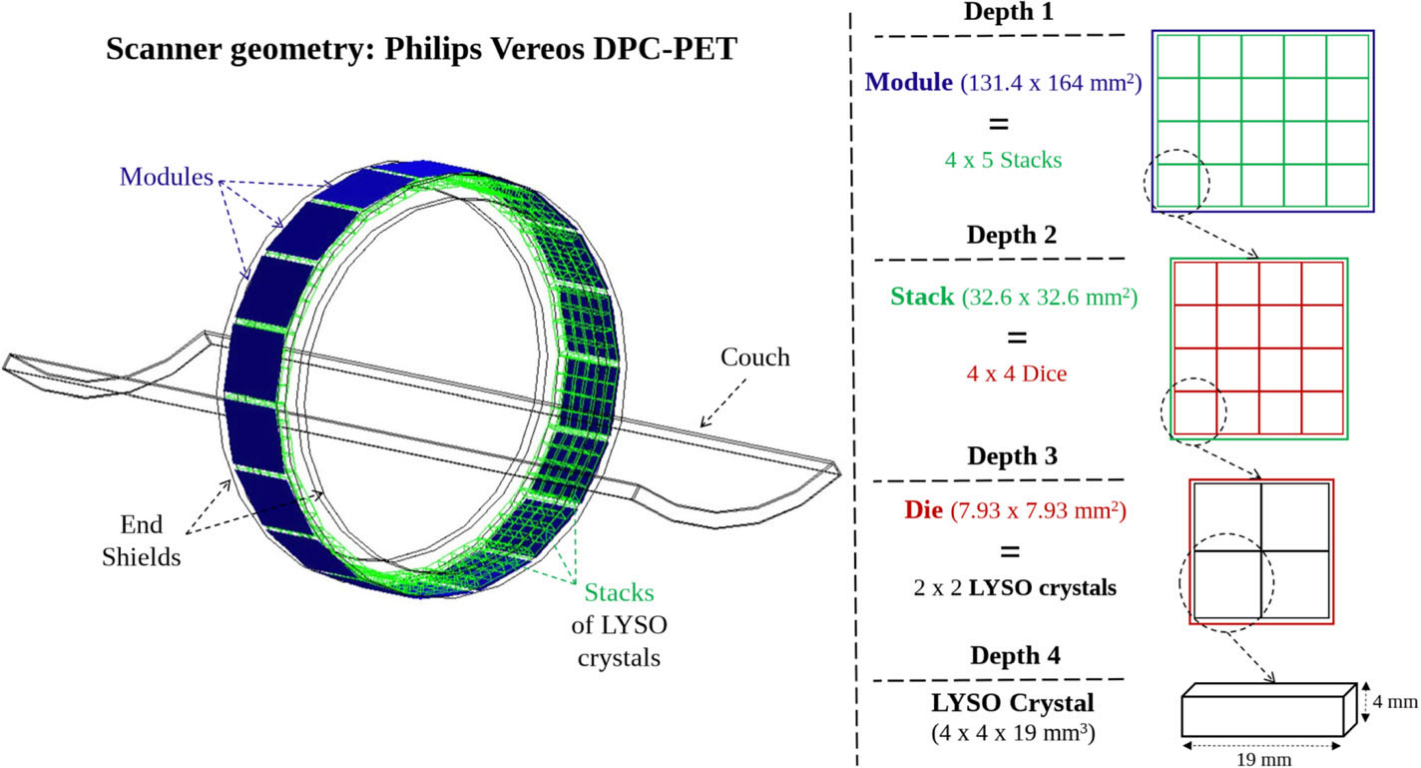
GATE geometry model of the Philips DPC-PET. The four depth levels components are illustrated on the right of the image. Eighteen modules are repeated in one ring. Each module contains 4 tangentially ×5 axially arranged stacks. Each stack is subdivided into 4×4 dice and each die contains 2×2 LYSO crystals. The system is composed of a total of 23 040 crystals

The geometry, dimensions, and material composition of the scanner were provided by Philips. The cylindrical PET was defined by a set of hierarchically arranged elements with four different depth levels. The first level component was called a *module*. Eighteen modules were arranged in one ring and each module was composed of an array of 4×5 blocks, called *stacks* (second level). The stacks were individually subdivided into 4×4*dice* (third level). Each die consisted of a grid of 2×2 lutetium–yttrium oxyorthosilicate (LYSO) scintillator crystal elements (4th level). Spacing and packing materials between the different detector blocks were taken into account. The final configuration leads to one detector ring with a total of 23 040 LYSO scintillator crystals, with individual dimensions of 4×4×19 mm^3^, resulting in an axial FOV of 164 mm and a detector cylinder of 764 mm inner diameter. Figure 1 shows the geometry of the PET model. In addition to the detector rings, the model included the lead shielding rings (which reduces the interference from activity outside the FOV), the inner diameter plastic cover and all the back compartments to take into account scatter in surrounding materials. The bed and different NEMA phantoms used were also modeled to take into account photon attenuation.

### Photon detection and coincidence events

In a PET imaging system, coincidence events are detected by scintillation detectors coupled with photo-detector systems. Incident annihilation photons of 511 keV interact in the inorganic crystal (here: LYSO) and generate, by scintillation, thousands of flash optical photons that are detected by the photo-detector (here: SiPM). Even if Monte Carlo tracking of optical photons can be performed [42], it would require a tremendous amount of computation time to perform a complete simulation, estimated to be three orders of magnitude longer than without optical photons. Instead, because the number of generated scintillation photons is proportional to the energy deposited in the crystal, an analytical model is used through a specific *digitizer module* that converts photon interactions in the crystal into digital counts and manages the timestamp of all events [13]. The digitizer is composed of successive signal processing operations that mimics the photo-detection process.

**Fig. 2:**
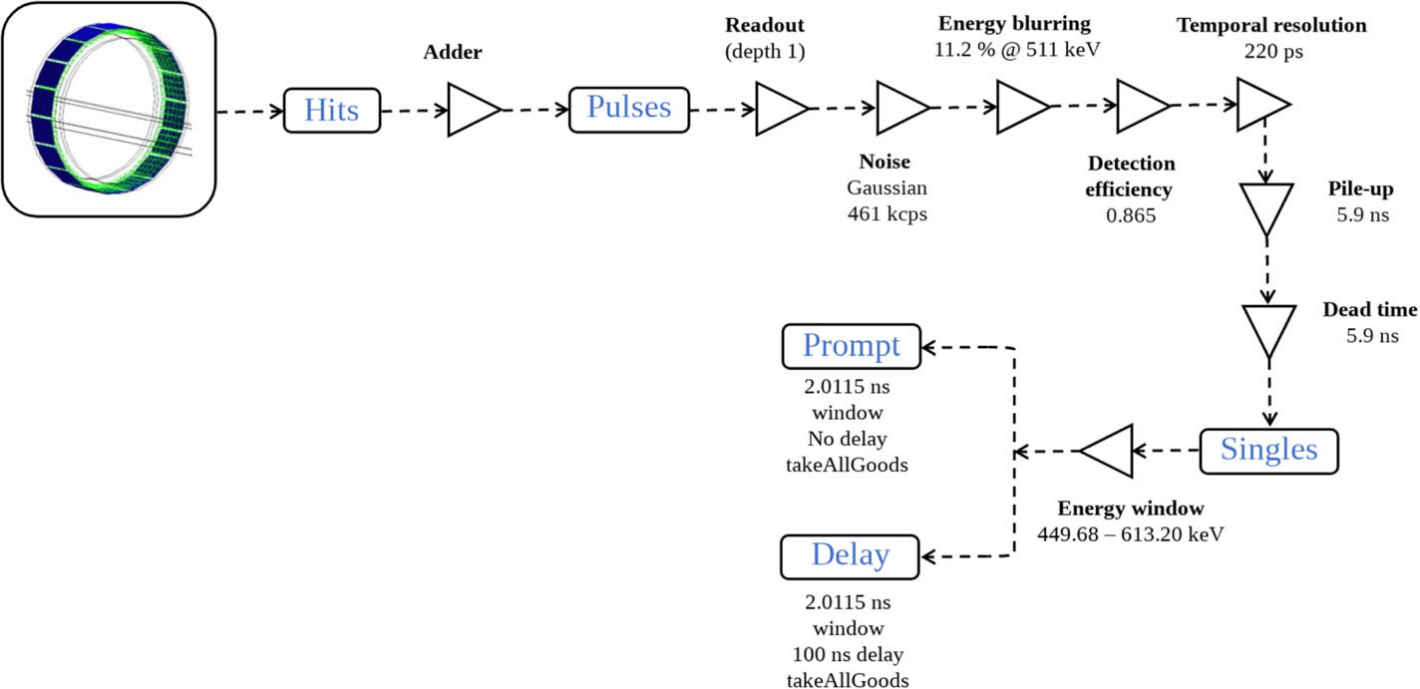
Digitizer chain used in GATE simulations

Figure 2 gives the schematic representation of the proposed signal processing chain. Individual particle interactions within the crystal are called *hits*. They are gathered into *pulses*, converted into *singles* and sorted into final *coincidences*. Along the chain, several models and parameters values have been chosen: (1) background noise, (2) energy resolution, (3) detection efficiency, (4) temporal resolution, (5) pile-up, (6) deadtime, (7) energy thresholds, and (8) coincidence window. Some parameters have been set according to constructor data, others according to a method adapted from Guez et al. [43]. They are described below and Table 1 depicts all parameter values.

**Table 1:** Parameter values of the digitizer chain

**Single event processing parameters**	
Background noise level	461 kHz
Crystal blurring	11.2% @ 511 keV
Detection efficiency	86.5%
Temporal resolution	220 ps
Pile-up	5.9 ps
Deadtime	5.9 ps
Energy window	449.68–613.20 keV
**Coincidence builder**	
Coincidence window	2.0115 ns
Delayed window offset	100 ns

#### Background noise, deadtime, pile-up, and detection efficiency

The background noise can be due to the electronics and the natural radioactivity of the crystals (^176^Lu in the LYSO crystals) and cannot be neglected at low activities below a few MBq. Background noise was modeled using stochastic energy, according to a Gaussian distribution, and inter-event time intervals, according to a Poisson process. The deadtime is the time after each event during which the system is not able to record another event. Pile-up events can occur when the, possibly partial, sum of the deposited energies from the two events is recorded as one single event instead of two separate events. The detection efficiency takes into account the quantum efficiency of the crystals and light transfer between the crystals and the SiPMs.

Following [43], all parameters have been determined from experimental measurements and simulations. First, we used the *singles* data from the phantom acquisition, as stated in the NEMA NU 2-2018 guidelines for measurement of the scatter fraction, count losses and randoms. For the low activity range, below 3 kBq mL ^−1^, linear regression was performed to estimate the theoretical *singles* rate in ideal conditions, in the absence of deadtime and pile-up influence. The fitted line was extrapolated to the origin to estimate the background noise level. The deadtime *τ* was then estimated using a paralyzable model, following the equation: $\tau = \frac {-1}{S_{\text {in}}}\ln \left (\frac {S_{\text {out}}}{S_{\text {in}}}\right)$ [44], where *S*_in_ is the estimation of the *singles* rate without the effect of deadtime and *S*_out_ is the measured *singles* rate from experimental data. Once background noise, deadtime, and pile-up have been modeled, the simulated and experimental single rates were compared by computing their ratio. This ratio should be constant over the entire range of activity concentration and provide the system detection efficiency. Final values were a frequency of 461 kcps for the noise, 5.9 ns for both the deadtime and pile-up values, and 86.5*%* for the detection efficiency. It should be noted that deadtime and pile-up were modeled before the energy windows and therefore their values differ from estimated experimental values.

#### Energy and temporal resolutions

Both the energy and temporal resolutions were set according to data from the manufacturer. The energy resolution was set to 11.2% at 511 keV for each crystal and the temporal resolution applied to *singles* was set to 220 ps leading to a coincidence temporal resolution of 311 ps.

#### Energy thresholds and coincidence window

As in the experimental setup, an energy window discriminator of 449.68–613.20 keV was used. The exiting *singles* were sorted by a *τ*=2.0115 ns coincidence timing window using the coincidence sorter module [45]. A second coincidence module was added with an offset of 100 ns in order to estimate the number of random counts in the simulation within a same window width. When more than two *singles* are detected in the coincidence window, several types of models could be used. Here, a *multiple-window* rather than a *single-window* approach was used to avoid counting some coincidences twice. The *takeAllGoods* multiples policy was selected. It considers all pairs of *singles* in the list of possible *coincidences* within geometric constraints. Also, this combination (*multiple-window* and *takeAllGoods*) provides a better estimation of the number of *randoms* using the delayed window [45]. As described by Moraes et al. [46], the design choices currently implemented in modern PET systems are best modeled in GATE by choosing the *takeAllGoods* coincidence policy.

### Image reconstruction

As explained earlier, the evaluation of the simulation was performed before image reconstruction to be independent of the algorithm, but examples of reconstructions were performed to illustrate image quality. For both reconstructed and simulated data, reconstructions were made with the open-source software CASToR (Customizable and Advanced Software for Tomographic Reconstruction) [47], using ordered subset expectation maximization (OSEM) [48] with Joseph line projector [49] (see below for detailed parameters).

### PET model validation

The proposed DPC-PET simulation model was evaluated following the NEMA NU 2-2018 [6]. The NEMA guidelines for PET provide a uniform and consistent method for experimental measurement and reporting performance parameters. The evaluation tests chosen for the validation were (1) count rates, NECR, and scatter fraction; (2) TOF resolution and energy resolution; (3) sensitivity; and (4) pre-reconstruction spatial resolution. The first three tests are independent of image reconstruction. For the spatial resolution, a LOR-based method was proposed in order to estimate spatial resolution from listmode data, before image reconstruction. Monte Carlo simulations were performed following the configurations described in the NEMA guidelines and compared to experimental measurements. Moreover, both experimental and simulated data were used to reconstruct images with the same reconstruction parameters to illustrate the image quality. Additional NEMA tests related to the accuracy of corrections and PET/CT registration accuracy were not performed.

#### Count rates, NECR, and scatter fraction

Scatter counts, C_sc_, is the number of falsely located coincidence events resulting from gamma rays scattering inside the phantom, C_*t*_ is the number of true coincidences, C_*r*_ is the number of random (accidental) coincidences, and C_tot_=C_sc_+C_*t*_+C_*r*_ is the total number of detected coincidences or *prompts*. Count rates are the counts divided by the acquisition time. Noise equivalent count (NEC) is a global measure of the scanner’s ability to detect useful counts, defined as $\textrm{NEC} = \frac{\text{C}_{t}^{2}}{\text{C}_\text{tot}}$, see for example [50]. Scatter fraction is defined as $\text{SF}=\frac {\text{C}_\text{sc}}{\text{C}_{t} +\text{C}_\text{sc}}$. NEC and SF evaluations were performed with a cylindrical phantom of 102 mm radius and 700 mm length, composed of polyethylene and centered in the FOV of the DPC-PET. The phantom was filled with a 1.6 mm radius line source of 1.78 GBq of ^18^F, off-centered at a radial distance of 45 mm from the central axis of the cylinder. Twenty-six different measurement time points were performed over 16 h and with increasing acquisition times. The activity during the last time point was less than 10 MBq. In order to reduce the computation time, the simulations were performed with the exact activity but with a reduced acquisition time such that each acquisition contained a minimum of 2×10^6^*prompts*. The experimental listmode data sets were truncated at 2×10^6^*prompts* for comparison with simulated data.

#### TOF and energy resolutions

The experimental and simulation setups were the same as for the previous test (the “Count rates, NECR, and scatter fraction” section). TOF resolution was determined for increasing count rates with the FWHM of the timing error histogram computed from the listmode data. This method is based on the work of Wang et al. [51] and was recently added to the NEMA NU 2-2018 [6]. There are two main differences compared to the daily quality control procedure, which uses a point source of ^22^Na. First, because of the 45 mm offset of the linear source used for the NECR measurement, the difference in detection time of coincident photons is no longer 0 and, therefore, it is necessary to compute a timing offset correction for each LOR, to account for the off-center source location. Second, scatter from the phantom must be corrected. The energy resolution was assessed with the FWHM value of the energy histogram computed from all true *coincidences* extracted from the same listmode data.

#### Sensitivity

Sensitivity was measured and simulated using a 700-mm-long and 1-mm-inner diameter line source filled with 8 MBq of ^18^F and centered in the FOV. Five acquisitions of 120 s were performed by placing the source successively inside one to five concentric aluminum sleeves of 1.25 mm thickness each. Sensitivity for each thickness was computed in cps MBq ^−1^ and the sensitivity without attenuation was extrapolated to zero thickness, to obtain effective sensitivity in the absence of scattering medium.

#### Intrinsic spatial resolution

Spatial resolution is usually determined after reconstruction, using the FWHM of the point spread function (PSF) of a point source. However, in order to remain independent of the reconstruction software for both experimental and simulation data, an original method was developed to estimate and compare spatial resolution from listmode data. Spatial resolution was assessed in the center of the axial FOV for five transverse positions, (0,1,0), (0,10,0), (0,20,0), (10,0,0), and (20,0,0) cm, using a 2.2 GBq mL ^−1^^18^F point source. Listmode data was acquired and simulated until a minimum of 3 million prompt events was reached for each position.

Due to the cylindrical shape of PET scanner, radial sampling increases with the distance from the center of the FOV. This non-uniform sampling in the transverse direction could lead to distortion of high spatial frequency and non-uniform spatial resolution across the FOV [52, 53]. Hence, listmode data was first resampled at a uniform sample rate of 2 LOR mm ^−1^ in the radial direction by the *arc correction* method [52, 53]. Then, for each event in the listmode, the orthogonal projection (A) of the point source (S) onto the LOR defined by the event was computed. The vector $\overrightarrow {SA}$, which represents the smallest vector between the LOR and the source, was projected along each axis *X*, *Y*, and *Z*. Each projected distance distribution along *X*, *Y*, and *Z* was then binned into histograms and the intrinsic spatial resolution for a given direction was determined by the FWHM. FWHM values were determined as preconized in the NEMA standard for the spatial resolution test [6]. This method requires a precise knowledge of the source position, which is the case for simulation but not for experimental data. The exact location of the point source was therefore determined by an optimization process aimed at minimizing the mean distance $\|\overrightarrow {SA}\|$ over all LORs.

In order to illustrate the difference between pre-reconstruction (intrinsic) and post-reconstruction spatial resolution, experimental, and simulated data of the central point source at (0,1,0) cm were reconstructed with 576×576 mm FOV, 1 mm voxels, using OSEM with 5 iterations and 10 subsets. Two reconstructions were performed. The first one with 4 mm isotropic Gaussian filter applied during the forward and backward model (the standard image-based PSF modeling), in order to match the FWHM of the DPC-PET at the center of the FOV [4] according to the NEMA standard (i.e., in air and with a filtered back-projection algorithm [6]). The second was performed with an additional 4 mm isotropic Gaussian filter applied to the final reconstructed image (the standard method of sieve). Spatial resolution was then determined using FWHM of the reconstructed PSF along the three main directions (axial, tangential, radial) as described in the NEMA.

#### Image quality

Illustrative reconstructed images were compared with the following protocol. A three minute PET acquisition of the International Electrotechnical Commission (IEC) torso phantom was performed. The phantom was filled with ^18^F of 5.3 kBq mL ^−1^ for the background activity and four times higher in four spheres of 10-, 13-, 17-, and 22-mm diameters. The 28- and 37-mm diameter spheres were left cold [54]. Images were reconstructed with OSEM using 10 subsets and 1 to 10 iterations. For experimental data, all corrections required for quantitative reconstruction (attenuation, scatter [55], random [56], normalization [57]) were precomputed outside CASToR using a software provided by Philips. For simulated data, only random and attenuation corrections were computed. Other corrections were not available. Consequently, all scatter events were discarded during reconstruction. The delayed window method was used to estimate random correction factors. An attenuation coefficient map was computed for the IEC phantom. Background relative noise (BRN) and contrast recovery coefficient (CRC) for all six spheres were computed as described in [58].

#### Implementation

In total, 36 PET listmode acquisitions were performed: 26 with the scatter phantom, 5 with the sensitivity analysis, and 5 with point sources for the spatial resolution estimation. All corresponding simulations were performed with the same set of digitizer parameters. Experiments have been done on a Philips Vereos system installed at Nancy University Hospital (Nancy, France). Simulations were conducted on a cluster with Intel Xeon CPU E5-2640 v4 @ 2.40 GHz, with 12 GB memory. The GATE macros used for the simulations are available on the website of the OpenGATE collaboration.

## Results

### Count rates, NECR, and scatter fraction

**Fig. 3:**
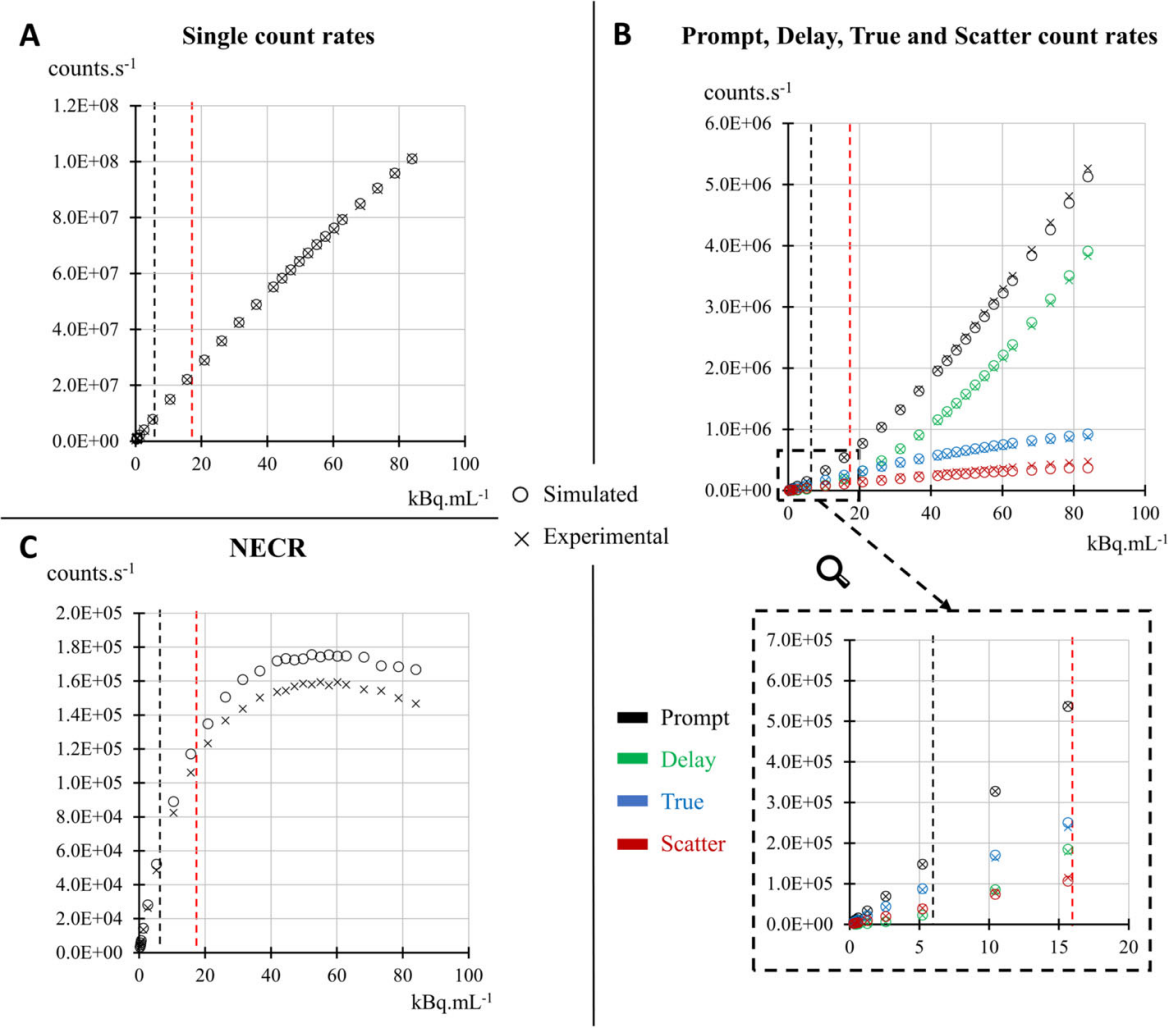
Simulated and experimental count rates according to activity concentrations. **a** Single count rates. **b** Prompt, delay, true, and scatter count rates. **c** NECRs. The vertical dashed lines correspond to the upper level of activity concentrations usually used in clinical routine for ^18^F (black line) and ^82^Rb (red line) PET exams

As depicted in Fig. 3a, a good agreement was obtained between simulations and experiments for *single* event rates, with a maximum of 0.7% relative difference up to an activity concentration of 80 kBq mL ^−1^. Note that usual activities used clinically with ^18^F-FDG tracer do not exceed 6 kBq mL ^−1^ [59]. Maximum relative differences between simulated and experimental data, up to 80 kBq mL ^−1^, were 3%, 3%, 5% and 18% for *total*, *random*, *true*, and *scatter* coincidence count rates, respectively (Fig. 3b). The difference for scatter count rate stayed below 5% up to a concentration of 10 kBq mL ^−1^.

As a result, less than 13% relative difference was obtained between simulated and experimental NECR (Fig. 3c). The peak NECR was close between simulated and experimental data with 175.6 kcps at 52.4 kBq mL ^−1^ and 159.4 kcps at 54.9 kBq‘mL ^−1^, respectively. At these peak values, the corresponding scatter fractions were found to be 29.2% and 33.2% for simulated and experimental data, respectively. Due to the systematic difference between simulated and experimental *scatter* events at high count rates, the simulated scatter fraction was also slightly underestimated, with a relative difference increasing from 5% at 4 kBq mL ^−1^ to 15% at 80 kBq mL ^−1^. However, this difference in *scatter* event rate only becomes important for activities that are much higher than those commonly used in routine clinical practice. Values for peak NECR and scatter fraction are reported in Table 2.

**Table 2:** Comparison of the characteristics of the DPC-PET for NECR and scatter fraction between the experimental and Monte Carlo simulated data in this study

Parameter	Experimental	MC simulation	Rausch et al. [4]	Zhang et al. [5]
NECR @ peak value	159.4 kcps @ 54.9 kBq mL ^−1^	175.6 kcps @ 52.4 kBq mL ^−1^	153.4 kcps @ 54.9 kBq mL ^−1^	171 kcps ^∗^ @ 50.5 kBq mL ^−1^
NECR @ 5.3 kBq mL ^−1^	48.5 kcps	52.2 kcps	47.2 kcps	n/a
Scatter fraction @ peak NECR	33.2% @ 54.9 kBq mL ^−1^	29.2% @ 52.4 kBq mL ^−1^	33.9% @ 54.9 kBq mL ^−1^	30.8% ^∗^ @ 50.5 kBq mL ^−1^
Scatter fraction @ low count rates	31.8% @ 0.4 kBq mL ^−1^	30.3% @ 0.4 kBq mL ^−1^	31.7% @ 0.4 kBq mL ^−1^	n/a

Additional comparison is added from experimental data published from Rausch et al. [4] and Zhang et al. [5]. Values marked with an asterisk (^∗^) represent ’maximum’ instead of peak values since the system did not show a peak value beyond which the NECR began to decrease with increasing activity

The maximum relative differences between simulated and experimental values, depicted in Table 3, were below 11% for all event rates as well as for NECR and scatter fraction, for activity concentrations of 2–6 kBq mL ^−1^ and 11–16 kBq mL ^−1^, which correspond to the clinical ranges found in ^18^F-FDG whole-body exams and in ^82^Rb cardiac exams [59], respectively.

**Table 3:** Maximum relative differences between simulated and experimental data are reported for activity concentration ranges for clinical ^18^F scans and ^89^Rb cardiac perfusion scans

	^18^F [2–6 kBq mL ^−1^]	^82^Rb [11–16 kBq mL ^−1^]
Single	− 0.6%	− 0.6%
Prompt	1.2%	− 0.5%
Delay	− 3.1%	− 3.4%
True	4.1%	4.8%
Scatter	− 4.7%	− 7.6%
Scatter fraction	− 5.8%	− 8.3%
NECR	7.5%	10.3%

### TOF and energy resolutions

**Fig. 4:**
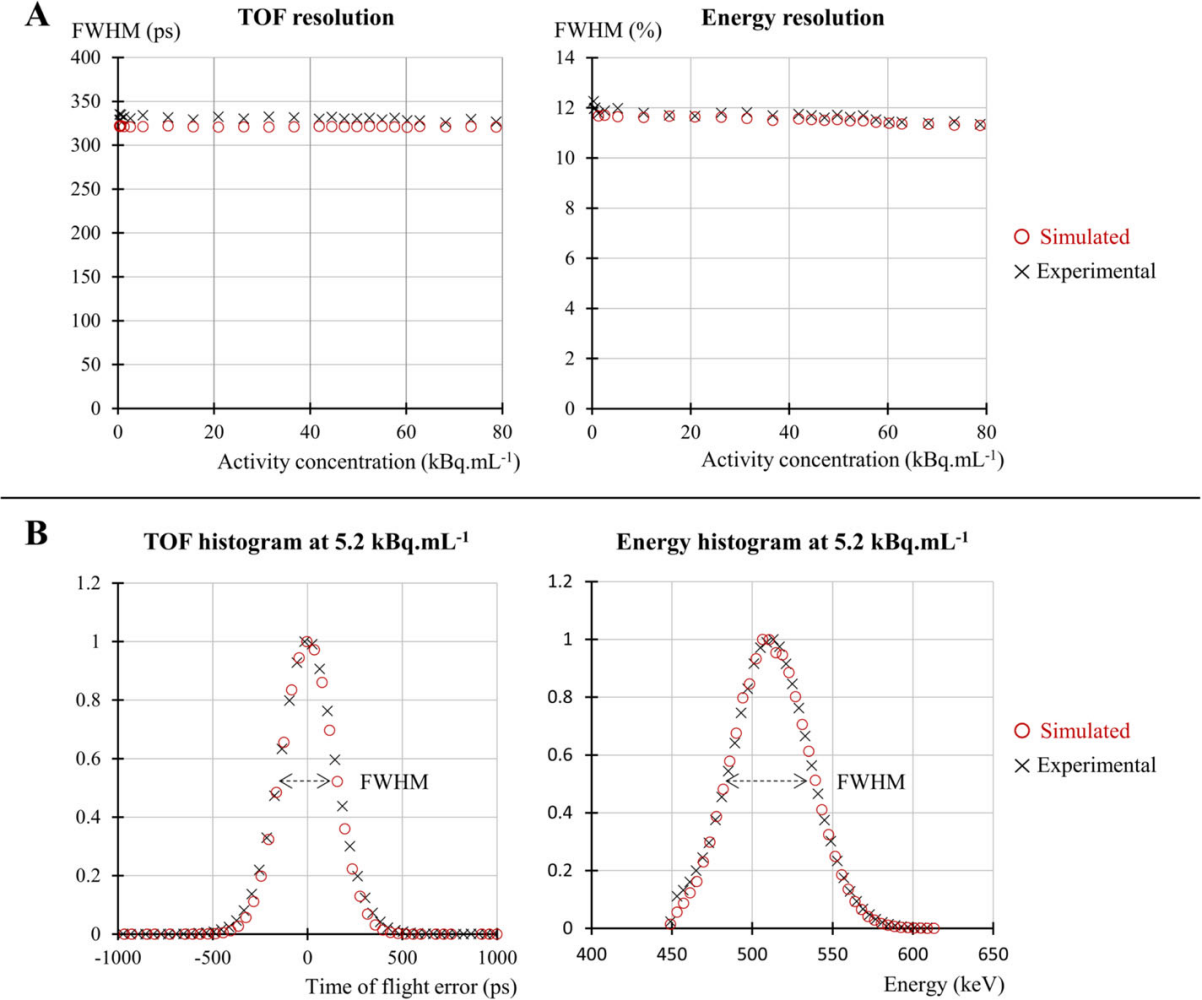
**a** TOF and energy resolutions according to activity concentrations for both simulated (o) and experimental (x) data. **b** Example, for 5.2 kBq mL ^−1^ activity concentration, of the normalized TOF and energy histograms used to compute the FWHM values

As depicted in Fig. 4a, a good agreement was obtained with less than 4% relative difference observed between simulated and experimental data for both TOF and energy resolutions over the entire range of activity concentrations explored in this study. Examples of TOF and energy histograms, from which the FWHM values were extracted, obtained for an activity concentration of 5.2 kBq mL ^−1^ are presented in Fig. 4b.

### Sensitivity

**Fig. 5:**
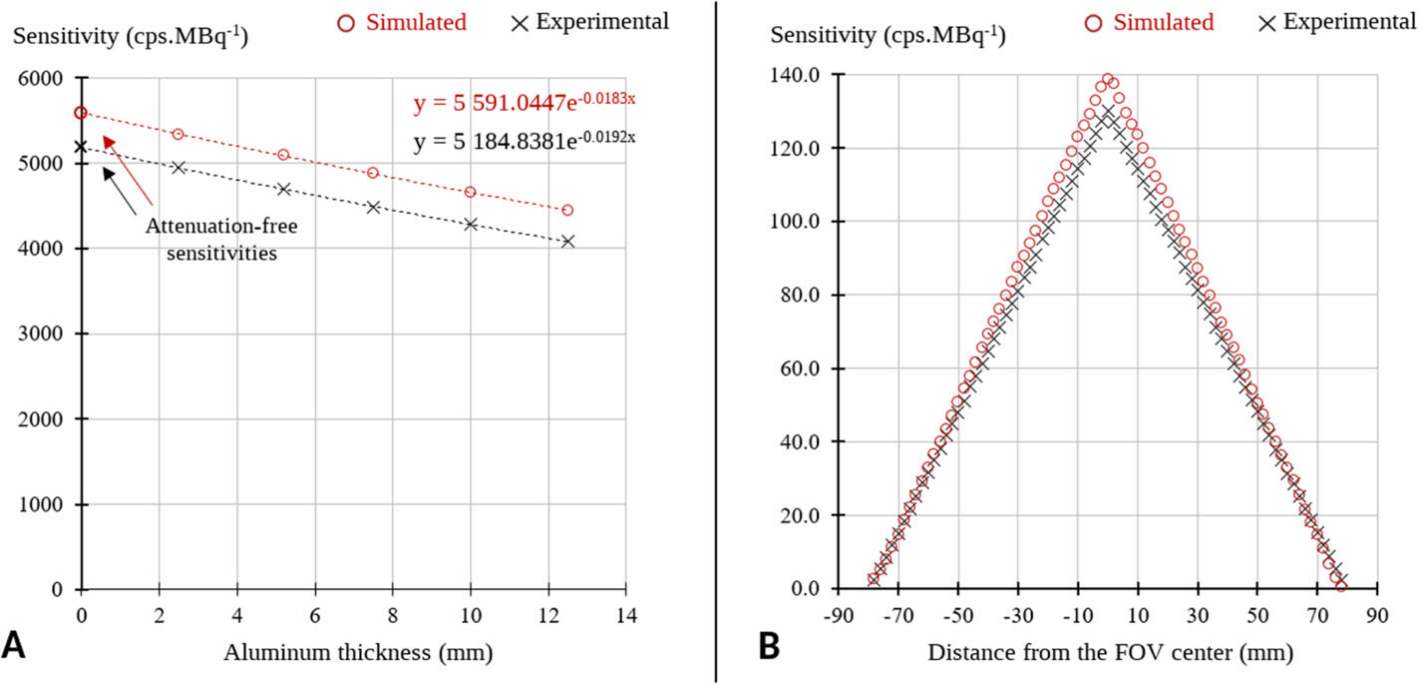
**a** Total count sensitivities measured according to NEMA standards for simulated and experimental data, for increasing thicknesses of aluminum sleeves and with the exponential regressions providing attenuation-free count sensitivities. **b** Sensitivities of contiguous axial slices according to the distances from camera centers

The measured and simulated sensitivities for each aluminum thickness together with the extrapolation for determining the attenuation-free sensitivity are provided in Fig. 5a. A 7.9% agreement was found between the attenuation-free sensitivity for simulated and experimental data, showing 5591 and 5184 cps MBq ^−1^, respectively. As shown in Fig. 5b, the axial sensitivity profiles between simulated and experimental data were in good agreement. Largest relative differences were within 14% for the low sensitivity slices on both sides of the FOV. Sensitivity decreases according to the distance from the camera center due to 3D acquisition geometry.

### Intrinsic spatial resolution

**Fig. 6:**
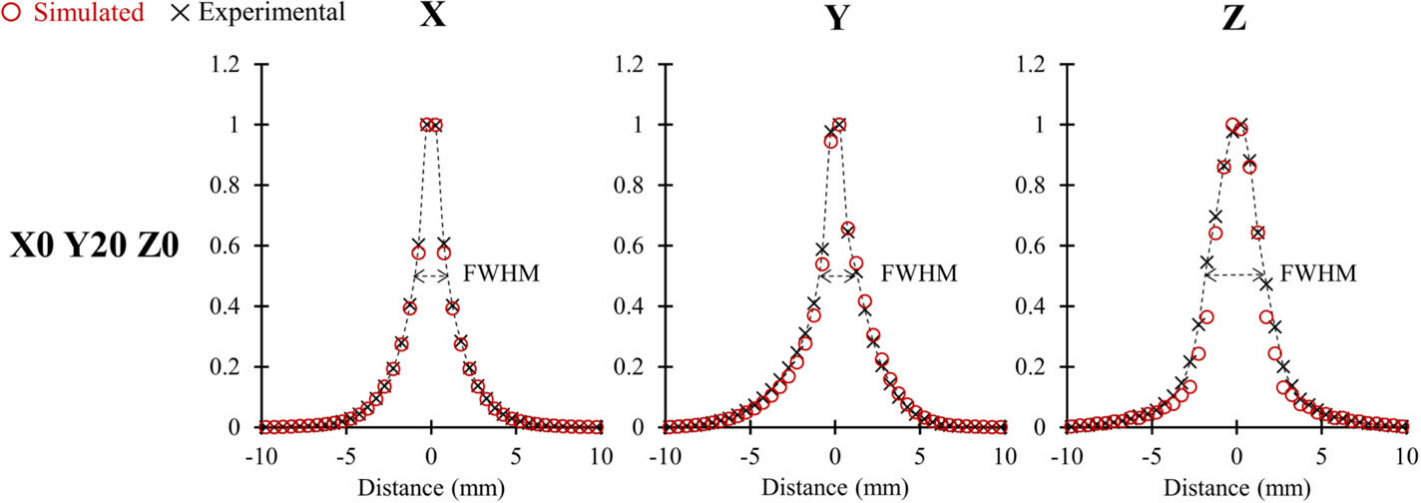
Example of histograms and corresponding FWHM of the shortest distances between source and LORs projected along the *X*, *Y*, and *Z* axis, obtained from experimental and simulated data

Table 4 depicts the FWHM for intrinsic spatial resolution for five transverse positions. An overall agreement of less than 0.25 mm absolute difference was obtained between simulated and experimental data, except for the *Z* axis (axial) where values up to 0.7 mm were obtained. Figure 6 provides an example of the histograms from which the FWHM were extracted for the (0,20,0) cm transverse position. Table 5 depicts the FWHM at the central position (0,1,0) cm obtained (1) before, (2) after reconstruction with image-based PSF modeling, and (3) after reconstruction with the standard method of sieve.

**Table 4:** FWHM defined before reconstruction for intrinsic spatial resolution along all dimensions for five transverse positions, for both simulated (Sim.) and experimental (Exp.) data

Position (cm)	Sim. FWHM (mm)			Exp. FWHM (mm)			Abs. diff. (mm)		
	*X*	*Y*	*Z*	*X*	*Y*	*Z*	*X*	*Y*	*Z*
(0,1,0)	1.73	1.48	1.91	1.93	1.71	2.37	− 0.20	− 0.23	− 0.46
(0,10,0)	1.87	1.97	2.81	1.97	2.09	3.51	− 0.10	− 0.12	− 0.70
(0,20,0)	1.77	2.19	2.99	1.90	2.18	3.37	− 0.13	0.01	− 0.38
(10,0,0)	1.82	1.69	2.76	1.95	1.87	3.50	− 0.13	− 0.18	− 0.74
(20,0,0)	1.77	1.73	3.01	1.89	1.90	3.40	− 0.13	− 0.17	− 0.40

The columns to the right give their absolute difference

**Table 5:** Comparison of the intrinsic FWHM obtained before reconstruction for the (0,1,0) cm central position, with FWHM obtained after reconstruction

Position (0,1,0) cm	Sim. FWHM (mm)			Exp. FWHM (mm)			Abs. diff. (mm)		
	*X*	*Y*	*Z*	*X*	*Y*	*Z*	*X*	*Y*	*Z*
Intrinsic	1.73	1.48	1.91	1.93	1.71	2.37	− 0.20	− 0.23	− 0.46
4 mm PSF	1.95	1.92	1.88	1.84	1.57	1.67	0.10	0.35	0.22
4 mm Sieve	4.37	4.37	4.31	4.45	4.46	4.33	− 0.08	− 0.09	− 0.02

### Image quality

**Fig. 7:**
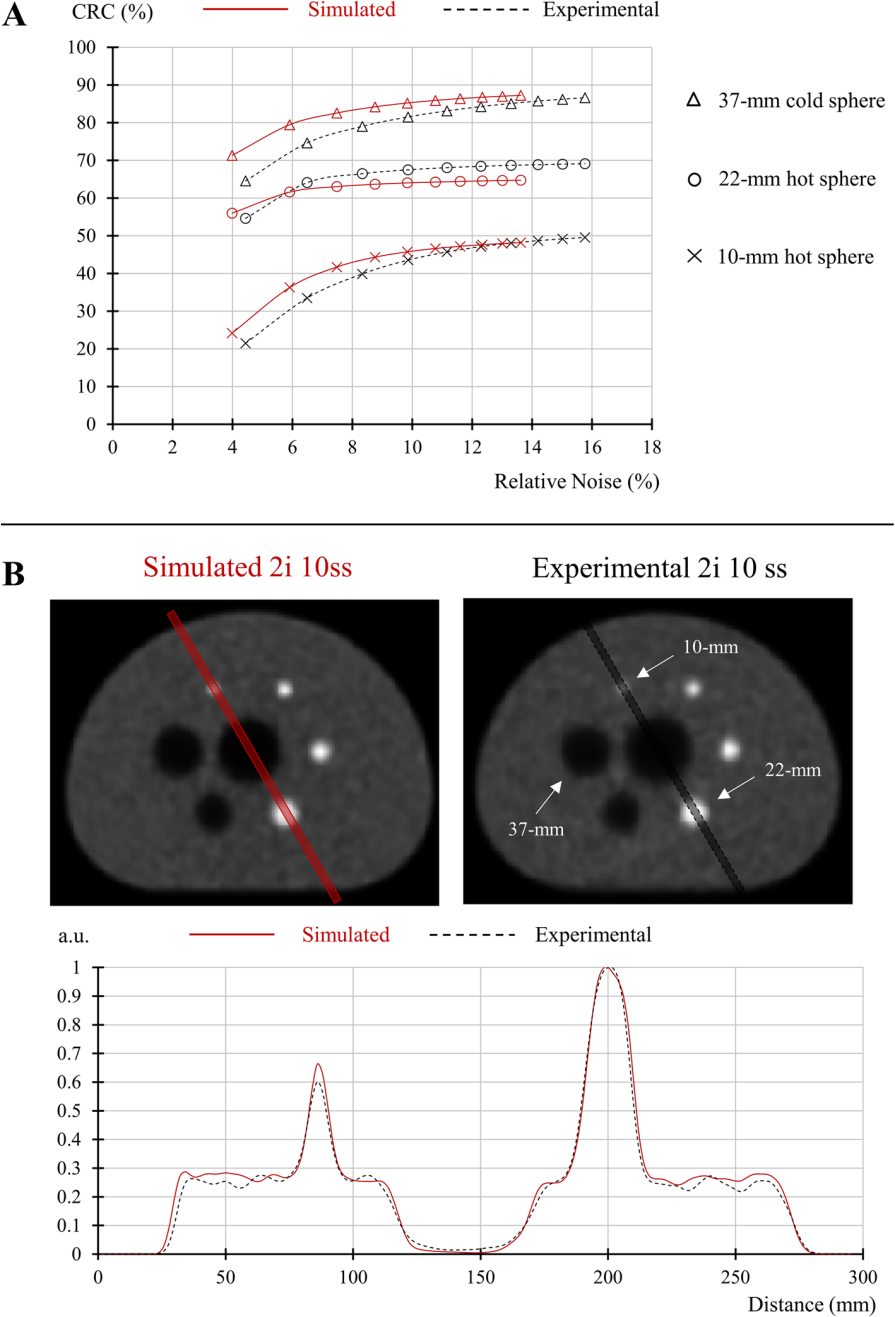
**a** Comparison between simulated and experimental images of the relationships between contrast-recovery-coefficients (CRC) and relative noise (RN), according to the number of OSEM iteration and for the 10- and 22-mm diameter hot spheres and the 37-mm cold sphere. **b** Cross-sectional slices passing through the spheres of the IEC phantom and reconstructed with 2 OSEM iterations and 10 subsets from simulated and experimental data. A parallelepiped profile of 6 mm cross-section and passing through the hot spheres of 10-mm and 22-mm diameter is compared between simulated and experimental images

The tradeoff between CRC and BRN was analyzed for two hot spheres (10 and 22 mm) and one cold sphere (37 mm) of the IEC phantom. Curves representing the evolution of CRC according to BRN as a function of the number of iterations are shown in Fig. 7a. In overall, the tradeoff was slightly better for simulated compared to experimental images. At early iterations, simulated images show better CRC with a maximum relative difference of 13% (at 1 iteration), while for further iterations, BRN is better with a maximum relative difference of 14% reached for 5 iterations. Figure 7b shows that simulated and experimental images were visually very close. The depicted profiles passing through both the 10 and 22 mm spheres were almost identical.

### Monte Carlo statistical uncertainty

In order to estimate the statistical uncertainty from the Monte Carlo simumations, 10 different simulations of the NEMA count rate test were performed at both 5.2 kBq mL ^−1^ and 42 kBq mL ^−1^, using 2×10^6^*prompts*. The coefficient of variation (ratio of the standard deviation to the mean) over the 10 values for the *true*, *scatter*, and *delay* event rates were calculated and reported in Table 6.

**Table 6:** Monte Carlo relative statistical uncertainty for the true, scatter, and delay event rates

	5.2 kBq mL ^−1^	42 kBq mL ^−1^
True	0.2%	0.3%
Scatter	0.4%	0.9%
Delay	0.2%	0.1%

## Discussion

In general, the proposed simulation model has been favorably compared to measurements. Excellent agreement was found between simulated and experimental *singles* count rates with relative differences below 1% over the whole activity concentration range. For *total*, *random*, and *true* coincidence rates, agreements were found with maximum differences equal to or less than 5% on overall. For *scatter* count rates, agreements reached a maximum of 18% difference for activity concentrations higher than 10 kBq mL ^−1^. The cause of this discrepancy at high activity is not yet explained; it might be due to elements which produce scattered radiation being included in the simulation by simplified geometrical models, such as the cooling plates, the Lexan cover, or the patient table. It could also be due to the simulated composition of the materials of the cylindrical phantom which might not be exactly the same as in the real phantom. However, for scatter fraction below 10 kBq mL ^−1^, the percentage differences were within 6% between simulated and experimental values and stayed within 15% for higher activity concentrations.

Experimental peak NECR was in agreement to the study of Rausch et al. [4] showing 3.8% and <1*%* differences for the NECR value and the corresponding activity concentration, respectively. The simulated peak NECR value was 175.6 kcps at 52.4 kBq mL ^−1^, which represents a relative difference from the experimental values of 10.2% for the peak NECR and 4.6% for the corresponding activity concentration.

As a consequence, the proposed model reproduces all experimental counting rates with less than 8% relative errors on the range of clinical activity concentrations found in ^18^F-FDG whole-body exams (2–6 kBq mL ^−1^) and less than 11% until the upper limit of 16 kBq mL ^−1^ found in the first pass (first 30 s) of dynamic ^82^Rb cardiac exams.

One of the main features of the Vereos DPC-PET, in comparison with conventional analog PET, is its good count rate performances characterized by low dead time and pile-up effects. This property is mainly due to the use of SiPM allowing a 1:1 coupling with the scintillation crystals. As a result, the relative difference between the theoretical and the experimental *singles* rate obtained by the low activity linear regression in the absence of dead time, was less than 5% up to an activity concentration of 15 kBq/mL. However, even if the loss due to dead time was relatively low with the Vereos DPC-PET, it was necessary to model this effect to obtain accurate counting rates, especially for high activity concentrations.

At clinical activity concentrations, background noise from electronics and natural radioactivity from crystals are often considered negligible and is thus not modeled in Monte Carlo PET simulations. However, it was necessary in order to reproduce the experimental low-activity counting curves. For example, at the lowest measured concentration of 0.3 kBq mL ^−1^, the background noise represented 60% of the total number of detected *singles*. This percentage decreased with activity concentration, but was still 6% at 5 kBq mL ^−1^ which is representative of clinical ^18^F-FDG activity. It dropped below 1% as from an activity concentration of 35 kBq mL ^−1^.

Figure 4a shows the stability of TOF and energy resolutions up to 80 kBq mL ^−1^. An agreement within 4% between both the simulated and experimental timing and energy resolutions was found over the whole activity range. Simulation values were slightly lower than experimental ones. The sensitivity value obtained by simulation (5591 cps MBq ^−1^) was found to be 7.9% higher than the experimental one (5184 cps MBq ^−1^). A good agreement was obtained between the axial sensitivity profiles (Fig. 5b), with a maximum difference of 13%. However, in comparison with published values from Zhang et al. (5721 cps MBq ^−1^) [5] and from the Philips white paper (5390 cps MBq ^−1^) [60], the agreement was closer with 2.3% and 3.7% relative differences, respectively. This disparity between the experimental sensitivity values could be explained by the uncertainty on the activity calibration as well as on the phantom positioning which is critical for this test.

The estimation of intrinsic spatial resolution before reconstruction was close between simulation and experimental data, with less than 0.7 mm absolute difference. Intrinsic spatial resolutions were better in the transverse direction than in the axial direction for both simulated and experimental data (see Fig. 6 and Table 4). This PSF asymmetry is inherent to the proposed method. Indeed, due to the cylindrical shape of the scanner, only a few azimuthal angles are available in the axial direction. The projected distance along *Z* can only take values between a scalar *b* and $\|\overrightarrow {SA}\|$, with *b* being the minimum value of the distance projected along the *Z* axis for a given distance $\|\overrightarrow {SA}\|$. The value of *b* depends on the axial position due to the truncation of the axial data in 3D PET. Therefore, the lowest projected distance values (between 0 and *b*) were not present in the distribution of the axial projected distance, resulting in a degraded FWHM in comparison with those measured in the transverse *X* and *Y* directions. In addition, the truncation of the projections also results in non-uniform axial sampling, making the resolution measurement inaccurate with the presented method for positions too far from the center of the axial FOV. Hence, the spatial resolution evaluation at positions located at 3/8 axial FOV from the center of the FOV, indicated in the NEMA protocol, were not performed in the present study. One solution to improve this method would be to use an exact rebinning algorithm, such as the one proposed by Defrise et al. [61], followed by resampling to achieve an axial sample rate comparable of that obtained transversely.

Regardless of the type of convolution chosen, good agreement was observed between simulated and experimental reconstructed resolutions with a maximum absolute difference of 0.35 mm. It is interesting to note that only when the convolution was applied in the reconstruction model and when the OSEM algorithm converged sufficiently (5 iterations and 10 subsets), that FWHM values were close to those obtained with the intrinsic resolution method developed. When post-reconstruction convolution filters were added, FWHM values were close to the manufacturer’s evaluation (4 mm).

Reconstructed images were in good agreement between the simulated and reconstructed data with a maximum relative deviation of 13% for the CRC and 14% for the BRN. The tradeoff between CRC and BRN was consistently slightly better for simulated than with experimental data. This difference can be explained by the scattered coincidences that have not been equally corrected: the experimental data were corrected by the standard single-scatter simulation (SSS) method [55], which only corrects for single-photon scattering, whereas the simulated data were reconstructed ignoring all scattered coincidences (ideal case).

Monte Carlo relative statistical uncertainty were lower than 1% on the estimated count rates. Simulation computation times were relatively large, with an average of about 3000 simulated primary *β*^+^ particles per second, leading to, for example, about 80 h of computation time for one of the 26 acquisitions with the scatter cylindrical phantom. No particular attention has been paid to gain speed. In particular, the slowest physic list (emstandard_opt4) has been used and the production and tracking cuts have not been optimized. The optimal trade-off between computation speed and accuracy remains to be studied, but this work can serve as reference for optimal accuracy results. Fast methods, such as the SMART software [62], may be used to speedup the simulation.

## Conclusion

As a conclusion, the proposed GATE PET model was validated with respect to NEMA compliant experimental data. To our knowledge, this is the first time a full Monte Carlo model of a DPC-based PET system is proposed and validated. This model can be useful for numerous studies such as to optimize imaging performance, evaluate reconstruction algorithms, and estimate the effects of confounding factors in image quality.

## Data Availability

The datasets used and/or analyzed during the current study are available from the corresponding author on reasonable request.

## Footnotes

1 See https://geant4.web.cern.ch/node/1731
